# Measuring the Environmental Burden of Disease in South Korea: A Population-Based Study

**DOI:** 10.3390/ijerph120707938

**Published:** 2015-07-13

**Authors:** Seok-Jun Yoon, Hyeong-Su Kim, Jongsik Ha, Eun-Jung Kim

**Affiliations:** 1 Department of Preventive Medicine, College of Medicine, Korea University, Seoul 136-705, Korea; E-Mail: yoonsj02@korea.ac.kr; 2 Department of Preventive Medicine, School of Medicine, KonKuk University, Seoul 143-729, Korea; E-Mail: mubul@kku.ac.kr; 3 Korea Adaptation Center for Climate Change, Korea Environment Institute, Sejong 339-007, Korea; E-Mail: jsha@jei.re.kr; 4 Department of Economics, Economic Research Institute, Korea University, Seoul 136-701, Korea

**Keywords:** environmental disease, environmental burden of disease, environmental risk factors, DALY

## Abstract

*Background:* This study attempted to measure the environmental burden of disease by examining mortality and disability rates in South Korea, permitting international comparisons. *Methods:* Disability-adjusted life years (DALY) was used to analyze data from public records. Years of life lost (YLL) and years lost to disability (YLD) were measured in terms of incidence rate and number of deaths. Attributable risks were based on those for WHO Western Pacific Regions. For air pollution, attributable risk was calculated using local PM_10_ levels and relative risk. *Results:* The total Korean environmental burden of disease was 17.98 per 1000 persons and the most serious risk factor was air pollution, at 6.89per1000 persons. Occupation was the second highest contributing factor, at 3.29 per 1000 persons, followed by indoor air pollution at 2.91 per 1000 persons. The DALY of air-pollution (indoor and outdoor) was 9.80 per 1000 persons, accounting for more than half of the total environmental burden of disease. The burden of chronic obstructive pulmonary disease, lung cancer, and asthma were 4.07, 3.16, and 1.96 per 1000 persons, respectively. *Conclusions:* Respiratory illnesses comprised most of the disease burden, the majority of which was linked to air pollution. The present results are important as they could be used to make evidence-based decisions regarding the management of diseases and environmental-risk factors.

## 1. Introduction

As correlations between pollutants and health are increasingly drawing social attention, efforts to evaluate the relationship between environmental pollution and diseases have been undertaken worldwide, such as the 2007 World Health Organization (WHO) report on environmental burden of diseases. The 2000 WHO report listed 13 environmental risk factors: outdoor air pollution, indoor air pollution, water, sanitation, hygiene, climate change, occupation, noise, other housing risks, recreational environment, land use and built environment, other community risks, and radiation [1,2]. These reports indicated that South Korea ranked 50 among 191 countries, with a score of 26 for disability-adjusted life years (DALY) per 1,000 persons. This was a much higher value compared to other WHO Western Pacific Regions such as Japan (15/1000), Singapore (14/1000), and Australia (16/1000). Thus, the present study addresses concerns regarding data-relevance.

Several studies on measuring the environmental burden of disease have been conducted; some evaluated the utility of research methodologies and their results [3] others gauged air pollution in urban areas [4] using Population Attributable Fraction (PAF) and PM_2.5_ concentration levels [5]. In addition, various studies have investigated the burden of disease according to environmental risk factors, such as water pollution [6] or climate change [7,8], while others investigated specific subjects such as regional residents, individuals in developing countries stratified by age [9,10,11], or comparisons between children and adults [12].

In Korea, Lee and colleagues have reported on pollution exposure and its effects [13], Jung and colleagues investigated the health condition of residents in mining areas [14], and Back examined the correlation between outdoor and indoor air pollution and asthma [15]. However, all Korean studies have only investigated the correlation between pollutants and health conditions or focused on patterns of diseases with prevalence or incidence rates as dependant variables. In other words, no study has measured the burden of disease by quantitatively analyzing risk factors. 

In this study, we calculated DALY for diseases with environmental risk factors-based on measured Korean public health data (as opposed to estimates), and assessed the impact of environmental pollution on health conditions by quantifying the burden of disease. 

## 2. Methods

### 2.1. Data Collection

Our study considered all ages of the Korean population in 2007. To investigate incidence as a measure of the burden of disease, we included first-time inpatients at hospitals in 2007. We assumed that individuals struggling with illnesses were likely to seek hospital treatment. We therefore excluded patients who visited hospitals in 2004–2006 for the same diagnosis as per the International Classification of Disease (ICD)-10 [17]. We also used Health Insurance Evaluation Review and Assessment Service (HIRA) insurance and claim data. 

Some diseases (e.g., asthma, chronic obstructive pulmonary disease (COPD), and diarrhea), would have often required primary treatment at outpatient clinics and may have been managed without hospitalization; we therefore included incidence from outpatient records. Typically, chronic asthma and COPD patients have longer hospital stays. Thus, we defined incidence cases as patients who had no record of outpatient visits in 2004–2006 but had visited outpatient clinics at least thrice in 2007. For acute diarrhea, individuals with three or more hospital visits in 2007 were included.

To collect data on mortality, the number of deaths was divided by causes of death based on ICD-10 codes according to age and gender from national statistics of death causes in 2007. For data on injuries, number of deaths and incidence were classified according to age and gender based on raw data from a 2005 study on damage performed by Korea Centers for Disease Control and Prevention (KCDC). Despite the time gap, we used data obtained in 2005, as research on trauma was not conducted in 2007. 

### 2.2. Environmental Burden of Disease

Data were analyzed in three steps to measure the environmental burden of disease in Korea. Diseases occurring due to environmental risk factors were first classified, and the 13 WHO risk factors were defined as potential causes of diseases. In the second step, attributable risks were determined to evaluate the degree to which these factors affected each disease. DALY was then calculated based on epidemiological indices related to each disease (e.g., incidence, mortality, and survival rates).

**Figure 1:**
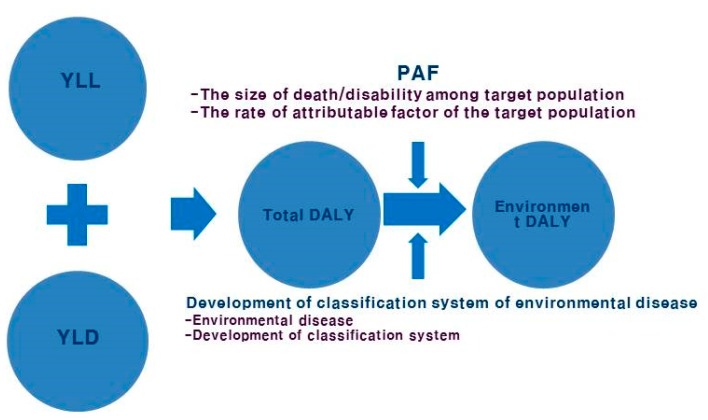
Research model for environmental burden of disease. DALY: disability-adjusted life year; PAF: population attributable fraction; YLL: years of life lost; YLD: years lived with disability.

To estimate the environmental burden of disease, we used DALY and PAF. The model structure for the environmental burden of disease is presented in Figure 1. To identify environmental diseases, those linked to the 13 WHO environmental risk factors and disease categories [1,2], were classified as per the ICD-10 code.

Regarding risk factors, we examined four major environmental risk factors identified by Valent and colleagues: indoor and outdoor air pollution, water pollution, and occupation [12]. We also considered the effects of climate change as they have increased considerably. Other risk factors including noise, other housing risks, chemicals, recreational environment, land use and built environment, other community risks, and radiation were also investigated to explore correlations between diseases and each risk factor.

To assess exposure to these 13 risk factors, attributable risk based on specific risk factors (*i.e.*, PAF) was measured. Korean data for air pollution using the PM10 standard [19] and water-pollution exposure based on water supply rates were used in this study. However, because of the lack of local data we used PAF reported by WHO for indoor air pollution, occupational factors, climate change, noise, other housing risk, chemicals, recreational environment, land use and built environment, other community risks, and radiation [18]. 

The burden of disease due to a specific risk factor—based on incidence and mortality rates—could be calculated by considering population exposure levels, its prevalence, age of onset, duration, and disability weight [16]. From these data, DALY could be obtained by adding years of life lost (YLL) and years lived with disability (YLD). We ensured comparability by applying a discount rate according to time value and age weights, as per WHO protocols. Therefore, by multiplying DALY with PAF of each risk factor according to disease, the environmental burden of disease—termed Korea-Environmental Burden of Disease (K-EBoD)—was calculated.

### 2.3. Statistical Analysis

The data used to measure burden of disease were processed with SAS 9.2 (SAS Institute, Cary, NC, USA). Basic data were derived for frequency analysis. In addition, DISMOD-II was used to calculate morbidity and average age of onset. The WHO has recently developed a newer version of DISMOD program (DISMOD-MR) for estimating the burden of diseases as a prevalence-based measure when population data is unavailable. However, since our study examines incidence-based burden of disease, we used DISMOD-II instead of DISMOD-MR.

## 3. Results

The database yielded by the three-step analysis was used to obtain DALY values. The burden of disease was calculated by multiplying DALY with PAF of environmental risk factors. For attributable risk of air pollution, regional PM_10_ levels based on the National Institute of Environmental Research (Ministry of Environment) data were used to determine the exposed population [19], and PAF was calculated using relative risks that were applicable to Korea based on a literature review [20,21,22,23,24]. PAF, derived from attributable risk, is presented inTable 1. 

**Table 1:** Attributable risk of diseases related to PM_10_ exposure.

Diseases	PAF
Preterm [20]	0.0679
Lung Cancer [21]	male 0.6837/female 0.2057
Chronic Obstructive Pulmonary Disease [22]	0.3024
Asthma [23]	0.1319
Ischemic Heart Disease [22]	0.3265
Pneumonia [24]	0.1340

To evaluate attributable risk of water pollution, exposure level was determined from water supply rates in Korea, which was 92.1% in December 2007 [19]. This study observed WHO guidelines for defining PAF [25], according to which, quality of water and hygiene could be divided into three categories: water supply, sanitation and hygiene, and water management and safety. The 11 diseases linked to these factors are presented in Table 2. In the present study, a scenario-based approach was used to calculate PAF of diarrhea; the Korean water supply rate was examined in relation to the relative risk measure proscribed by the WHO. The risk of diarrhea attributable to water pollution was 86%. For other diseases, attributable risks from WHO Western Pacific Regions were used. PAF levels [18] are presented in Table 3.

**Table 2:** Diseases related to water pollution and attributable risk.

Diseases and Attributable Risk	
Disease	Attributable Risk(%)
Diarrhea	86
Malnutrition	50
Intestinal nematode infections	100
Schistosomiasis	100
Trachoma	100
Lymphatic filariasis	82
Onchocerciasis	10
Dengue	95
Japanese encephalitis	95

PAF of each risk factor according to disease obtained is shown in Table 3 [25]. We used the PAF of developed countries in the Western Pacific region.

**Table 3:** Attributable risk factors according to diseases [25].

Risk Factor	Diseases ^a^	PAF of Developed Countries in Western-Pacific Region
Indoor air pollution	RI	lower: 0.20/upper: 0.12
	Perinatal conditions	0.06
	Lung cancer	male: 0.30/female: 0.30
	Cataracts	0.07
	COPD	male: 0.27/female: 0.09
	Asthma	0.44
Climate change	Diarrhea	0.90
	Malaria	0.40
	Dengue	0.95
Occupational factor	Lung cancer	0.18
	COPD	0.03
	Asthma	0.12
	Cardiovascular disease	0.12
	TB	0.19
	Cataracts	0.07
	Other unintentional injuries	0.08
	Neuropsychiatric disorder	0.01
	Other cancer	0.02
	Musculoskelectal disease	male: 0.17 / female: 0.20
	STDs	0.23
	HIV	0.08
	Hepatitis B, C	0.23
	Congenital abnormalies	0.23
	Falls	0.12
	Drowning	0.12
	Road traffic injuries	0.01
Other housing risks	Other unintentional injuries	0.08
	Falls	0.12
	Violence	0.16
Noise	Hearing loss	Male: 0.09 / female: 0.06
Chemicals	Congenital abnormalies	0.23
	Poisonings	0.71
	Suicide	0.001
Recreational environment	Other unintentional injuries	0.08
	Drowning	0.12
Land use and built environment	Other unintentional injuries	0.08
	Road traffic injuries	0.16
Water resources management	Malaria	0.40
Other community risks	Other unintentional injuries	0.08
	Falls	0.12
Radiation	Other unintentional injuries	0.08
	Congenital abnormalies	0.23

**^a^** RI = respiratory infection; COPD = chronic obstructive pulmonary disease; TB = Tuberculosis; STDs = Sexually Transmitted disease; HIV = Human Immunodeficiency Virus.

**Figure 2:**
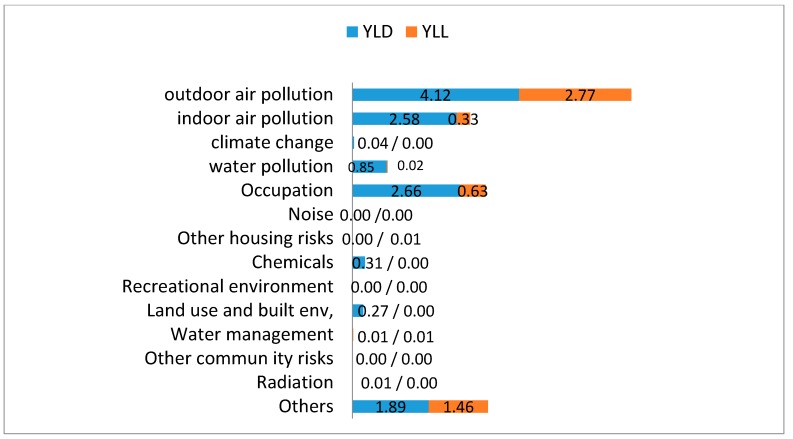
A composition of years lost to disability (YLD) and years of life lost (YLL) according to risk factors (unit: DALYs/1,000 persons).

As shown in Figure 2, the total K-EBoD was 17.98 per 1000 persons. Air pollution accounted for the largest proportion at 6.89/1000, followed by occupation at 3.29/1000, and indoor air pollution at 2.91/1000. When indoor and outdoor air-pollution values were combined, the burden of disease was more than half of the total K-EBoD, at 9.80/1000. The highest burden of disease was related to respiratory organs; COPD, lung cancer, and asthma were 4.07, 3.16, and 1.96 per 1000 persons, respectively. Considering that outdoor and indoor air pollution occupied the largest proportion of the burden of disease among risk factors, the results are unsurprising. The classification of the environmental burden of disease according to YLD and YLL is shown in Figure 2.

Diseases with a large portion of YLL could be considered as diseases with high mortality. YLD and YLL for outdoor air pollution was 4.12 and 2.77, for indoor air pollution was 2.58 and 0.33, and for occupation was 2.66 and 0.63 per 1000 persons.

Although the difference in burden of disease due to indoor air pollution and occupation was not large (indoor air pollution: 2.91 per 1000 persons; occupation: 3.29 per 1000 persons), the latter was considered more critical because of its relatively higher YLL.

This population-based study does not consider assumed values but measures actual values; we were therefore unable to suggest confidence intervals or uncertainty intervals.

## 4. Discussion

This study measured the environmental burden of disease in South Korea by evaluating the contribution of diseases with environmental risk factors to overall risk. 

We used the PAF of developed countries in WHO Western Pacific Region for environmental risk factors besides air pollution [18]. Classifying Korea as a developed or developing country was difficult. According to the Organisation for Economic Co-operation and Development (OECD), while South Korea ranked 10th in the economic scale among the 30 member countries, it ranked 17th for income disparity and 20th for average national income [26]. However, when WHO standards for classifying developing and developed countries are used—mortality rates of adults and of children aged at most 5 years—they were comparable to developed countries; the mortality rate of children in Korea (six deaths per 1000 persons) was not significantly higher than that of Japan (four deaths per 1000 persons), New Zealand (six deaths per 1000 persons), and Singapore (three deaths per 1000 persons). Therefore, attributable risk was calculated by classifying Korea as a developed country in the Western Pacific region. 

To evaluate exposure to the risk factors, attributable risk for specific risk factors was measured. Korean data for air pollution (using PM_10_ level) [19] and water pollution exposure levels (using water supply rate) were used. National statistics on exposure levels for indoor air pollution, occupational factors, climate change, noise, housing risks, chemicals, recreational environment, land use and built environment, other community risks, and radiation were either unavailable [18]. Or were not regarded as serious risk factors. This suggests that the government needs to prepare for the “global warming era” by collecting quantitative data to assess country-specific issues.

For COPD and asthma—with strong links to air pollution—YLD values in relation to DALY were 3.73 (4.07 DALY/1000 persons) and 1.85 (1.96 DALY/1000 persons), respectively. That is, their YLD was considerably higher than YLL. However, since quality of life is regarded as an important index of health, YLD should be treated seriously. Various policies need to consider support to manage illnesses. Moreover, the economic burden should be quantified and addressed. For example, the Korean Academy of Tuberculosis and Respiratory disease reported that medical costs for a patient with stage I COPD was about 1.39 million Won (KRW) per year, while that for stage IV was 3.13 million KRW, based on a nation-wide survey of eight hospitals. The morbidity of mild COPD was 17% in adults aged 45 years or older, and 41.4% in the elderly aged 75 years or older, indicating that the physical and economic burden on elderly individuals is huge [27].

However, examining economic burden was beyond the scope of this study. Moreover, it varied considerably between studies on the burden of disease. The WHO-CHOICE program that evaluates both the burden of disease and intervention costs could measure cost per DALY. Consequently, it would be possible to determine the efficacy of intervention programs for environmental risk exposure and compare their cost effectiveness. 

Although the study attempted to examine actual data on prevalence and mortality to measure DALY, such that it realistically reflects the realities of Korea, patients who did not visit a health facility during the study period were excluded. This may explain why the environmental burden of diseases was the lowest for the lowest economic group [28]. Some diseases such as vector-borne diseases, trauma, and occupational hazards were difficult to investigate. For trauma, data on discharged trauma patients were used, but for the other two HIRA data was utilized. While it was appropriate to use the following sources to measure DALY comprehensively, it did not meet various requirements. Regarding vector-borne diseases, statistics from the Center for Infectious Disease, KCDC, would have yielded accurate measurements. However, while it showed prevalence, it did not consider incidence for specific periods and therefore would not yield accurate DALY. Further, although specific measures from data on industrial accidents are recommended, Korea Workers’ Compensation and Welfare Service insurance and compensation statistics did not report incidence and disease onset, and was therefore considered inappropriate to measure occupational diseases in this study. 

In addition, although the outdoor air pollution risk factor was PM_10_ in this study, a more accurate measure of the burden of disease would be PM_2.5_, which includes fine particles and gaseous materials (NO_x_, SO_x_, CO, O_3_, *etc.*). Considering the possible harmful effects of PM_2.5_, the United States, European Union, and WHO have revised their recommended air pollution criteria (for example, the U.S. used ultrafine particles as a criterion of atmospheric environment). Korea has also attempted to include PM_2.5_ as a new criterion to improve atmospheric environment [19].

## 5. Conclusions

This study was the first to analyze recorded data in order to measure the environmental burden of disease in South Korea. Risk factors that significantly contributed to the burden of disease were evaluated and their effects were quantified by examining patterns. In particular, respiratory diseases accounted for the largest proportion and most diseases were attributed to air pollution. Exposure to pollution in was difficult to control, for example, air pollution is linked to other environmental factors, such as climate change affected by global warming, which leads to acid rain that affects soil acidification and water pollution. Because its effects are large-scale, prevention of air pollution should be considered a critical issue. In addition, smoking is an avoidable risk factor comprising a large proportion of indoor air pollution. The results are significant as they provide evidence-based information for decision-making regarding the management of diseases and environmental risk factors and for the development of environmental protection policies.

## Key Points

This study assessed risk factors that contributed to the burden of disease in Korea. Their effects quantified by calculating PAF and burden of the diseases according to risk factors. Respiratory diseases accounted for the largest portion. The causes of most diseases were attributed to air pollution. Moreover, it was difficult to avoid exposure to air pollutants. Due to global warming and the resulting climate change, air pollution leads to acid rain, increasing the acidification of soil and water pollution levels.

## Declarations

Funding: This work was supported by the Korea Health Industry Development Institute (Grant No. HI13C0729) and the National Research Foundation of Korea Grant funded by the Korean Government (NRF-2013S1A5B8A01054750).

Ethical approval: Approval for this study was obtained from the Korea University Institutional Review Board (Approval No. 1040548-KU-IRB-13-164-A-1(E-A-1)). Any identifying information from data and patients’ records were removed prior to analysis to ensure confidentiality.
